# Crystallization of Poly(3-hexylthiophene) Nanofiber in a Narrow Groove

**DOI:** 10.3390/polym8060231

**Published:** 2016-06-15

**Authors:** Satoshi Kushi, Ryota Tsukada, Keiichi Noguchi, Takeshi Shimomura

**Affiliations:** 1 Graduate School of Engineering, Tokyo University of Agriculture and Technology, Koganei, Tokyo 184-8588, Japan; ksks31044103@gmail.com (S.K.); tsukaryo1205@gmail.com (R.T.); 2 Instrumentation Analysis Center, Tokyo University of Agriculture and Technology, Koganei, Tokyo 184-8588, Japan; knoguchi@cc.tuat.ac.jp

**Keywords:** polythiophene, nanofiber, whisker, orientation, conductivity

## Abstract

Whisker-type poly(3-hexylthiophene-2,5-diyl) (P3HT) nanofibers were aligned by restricting their growth direction using an approximately 100–1000 nm wide narrow groove fabricated by thermal nanoimprinting. In grooves made of an amorphous fluoropolymer (CYTOP™) with widths of less than 1500 nm, the nanofibers oriented uniaxially perpendicular to the groove and their length was limited to the width of the groove. This result indicates that the nucleation of nanofibers tends to be selectively promoted near the interface of CYTOP™ with fluoro-groups, and nanofiber growth perpendicular to the wall is promoted because P3HT molecules are supplied more frequently from the center of the groove. Furthermore, the orientation induced anisotropic conductivity, and the conductivity parallel to the oriented nanofibers was more than an order of magnitude higher than that perpendicular to the oriented nanofibers.

## 1. Introduction

Molecular electronics, which are positioned to be the bottom-up technology, have attracted significant interest because they are constructed via a self-assembly approach, which allows for the possible elimination of a vast number of lithography steps [1,2]. Conducting polymers are one of the up-and-coming materials for molecular wires and molecular functional elements in molecular devices. They have properties suitable for the building blocks of molecular devices, such as a linear figure, high conductivity with doping, and semiconductivity. However, conducting polymers have some problems with respect to application as molecular wires. In particular, the carrier mobility of conducting polymers is much lower than that of inorganic semiconducting materials [3].

The carrier transport properties of conducting polymers are strongly affected by the degree of crystallinity and chain orientation. One effective approach for improving the degree of crystallinity is to use a whisker-type nanofiber structure made by crystallization in an adequate solvent. This method has been intensively investigated, particularly with regioregular poly(3-hexylthiophene-2,5-diyl) (P3HT) [4]. These P3HT nanofibers have been reported to have diameters of *ca.* 10 nm, in addition to a higher degree of crystallinity and a higher carrier mobility than conventional P3HT films [4,5,6,7,8,9,10,11,12,13,14]. However, the carrier mobility of this polymer nanofiber has remained significantly inferior to that of other inorganic semiconductors. Thus, an effective approach for improving the nanofiber orientation is essential to increasing the carrier mobility in conducting polymer nanofibers.

The electrospinning (ES) method can be used to align P3HT nanofibers uniaxially by a collector/electrode modification [15,16,17] or a collector drum rotation [18], and field-effect transistor (FET) measurements of aligned P3HT nanofibers have been performed [15,17]. However, P3HT nanofibers formed by this method did not have a sufficiently higher crystallinity than that of P3HT whisker-type nanofibers. On the other hand, a magnetic field and liquid crystalline orientation field have been used to orient whisker-type P3HT nanofibers [19,20]. Furthermore, off-central spin-coating has also been reported to uniaxially align the pre-aggregated fibrous structure of a conducting polymer [21]. The optical and electrical characteristics of aligned P3HT nanofibers obtained using these methods have been reported [19,20,21]; however, further exploration is required to achieve high performance in various applications.

In this study, whisker-type P3HT nanofibers were crystallized in a narrow groove with a width of approximately 100–1000 nm to align nanofibers by restricting their direction of growth. The preferential orientations for polymer crystals of syndiotactic polystyrene (sPS) [22], polyethylene oxide (PEO) [23,24], and poly(ε-caprolactone) (PCL) [25] in a confined space using the cylindrical nanodomains of an anodic aluminum oxide (AAO) template have been reported. The same effect is expected to appear for P3HT nanofibers crystallized in a narrow groove. P3HT is a conducting polymer; therefore, the preferential orientation of the P3HT nanofiber leads to anisotropic electrical conduction, which is not observed in conventional polymers such as sPS, and is thus the purpose of this study.

## 2. Experimental Section

### 2.1. Nanofiber Formation

Regioregular P3HT (*M*_w_ = 87,000) was purchased from Sigma-Aldrich Co., Inc. (St. Louis, MO, USA) and used without further purification. In our previous report, the nanofiber was crystallized from a solution of approximately 0.05 wt % P3HT in a mixture of anisole and chloroform as the solvent [11]. In this study, a denser 0.5 wt % P3HT solution in *m*-xylene was used because nanofiber crystallization in a narrow groove requires a small volume of solution and the solvent evaporates immediately. The P3HT solution was prepared by stirring at a high temperature (*ca.* 70 °C). After the reflux was stopped, the solution was immediately introduced by capillary flow into the narrow grooves on the substrate, which was covered with a glass slide. The sample was then cooled to −15 °C to crystallize the nanofibers. The nanofibers were used in the undoped state.

### 2.2. Preparation of Nanogrooves

The narrow grooves were fabricated by thermal nanoimprinting [26], as follows. An amorphous fluoropolymer, CYTOP™ (CTL-809M, Asahi Glass Co., Ltd., Tokyo, Japan), or a conventional amorphous polymer, poly(methyl methacrylate) (PMMA; Sigma-Aldrich Co., Inc., St. Louis, MO, USA, *M*_w_ = 120,000), was spin-coated at 3000 rpm onto a Si substrate with a 255 nm thick thermally grown SiO_2_ layer. A mold (NIM-100L RESO convex Ni, NTT-AT Co., Kawasaki, Japan) and the substrate were heated at 170 °C (CYTOP™) or 135 °C (PMMA), and the mold was pressed onto the polymer for 5 min at a pressure of 4.5 MPa using a thermal nanoimprinter (Nanoimpro^®^ Type105, Nanonics Co., Yonezawa, Japan). After gradually cooling to room temperature and demolding, the residual layer was completely removed by reactive ion beam etching (RIBE; EIS-200ER, ELIONIX Inc., Tokyo, Japan) with O_2_ gas for 8 s (CYTOP™) or 12 s (PMMA), according to the etching rates determined from preliminary measurements.

**Figure 1:**
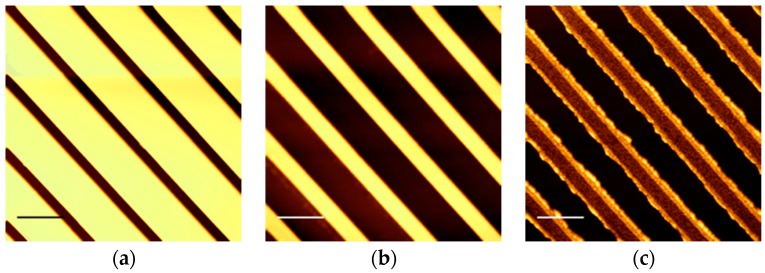
Scanning probe force microscopy (SFM) images of (**a**) the mold pattern; (**b**) the imprinted CYTOP^TM^ pattern; and (**c**) the pattern after removal of the residual layer. Scale bar represents 2.0 μm.

The width of the groove varied from approximately 600 nm to 3 μm and tended to be slightly larger than that of the mold due to the residual layer etching process. The height of the wall was approximately 100 nm. Figure 1 shows scanning probe force microscopy (SFM) images of the mold pattern, the imprinted pattern, and the pattern after removal of the residual layer.

### 2.3. Characterization of Nanofiber Orientation 

The groove pattern and the crystallized nanofiber in the pattern were observed using SFM (Nanocute, SII Nanotechnology Inc., Tokyo, Japan) in intermediate tapping mode. To analyze the nanofiber orientation in the SFM images, we calculated the autocorrelation images as follows. Initially, the SFM images of the nanofibers observed in the groove were clipped out and binarized with an appropriate threshold. Autocorrelation images were then generated using image analysis software (NIH Image, National Institutes of Health, Bethesda, MD, USA). Finally, the autocorrelation images observed at 6–11 different locations were averaged to generate an averaged autocorrelation image.

Wide angle X-ray diffraction (WAXD) measurements were performed using an X-ray diffractometer (SmartLab, Rigaku Co., Tokyo, Japan). The nanofiber for out-of-plane (θ/2θ) and in-plane (2θ_χ_/φ) measurements was crystallized at −15 °C on a nonreflecting Si plate (Overseas X-ray Service Co., Ltd., Saitama, Japan) that was covered by a thin film of CYTOP™ and then dried. In the in-plane measurements, the grazing incident angle *ω* and diffraction angle 2θ were fixed at 1.0° and 2.0°, respectively, and ω was slightly larger than the critical angle of the non-fibrillar P3HT film.

Conductivity measurements were carried out using a two-probe method in a vacuum below 10^−5^ Torr using a source/measure unit (SMU; 6430, Keithley Instruments, Inc., Cleveland, OH, USA) in a cryogenic probing station (LIPS, Nagase Techno-Engineering Co. Ltd., Tokyo, Japan). Pt electrodes were sputter-deposited through a metal mask by RIBE, and the width and gap of the electrodes were 40 and 600 μm, respectively. To investigate the anisotropic conduction, electrodes were deposited parallel and perpendicular to the grooves. The electrodes perpendicular to the groove were formed using an oblique deposition method to deposit metal on the groove wall, which prevented division of the electrode into isolated segments by the grooves.

## 3. Results and Discussion

### 3.1. Nanofiber Growth in Grooves

#### 3.1.1. CYTOP^TM^ Grooves

**Figure 2:**
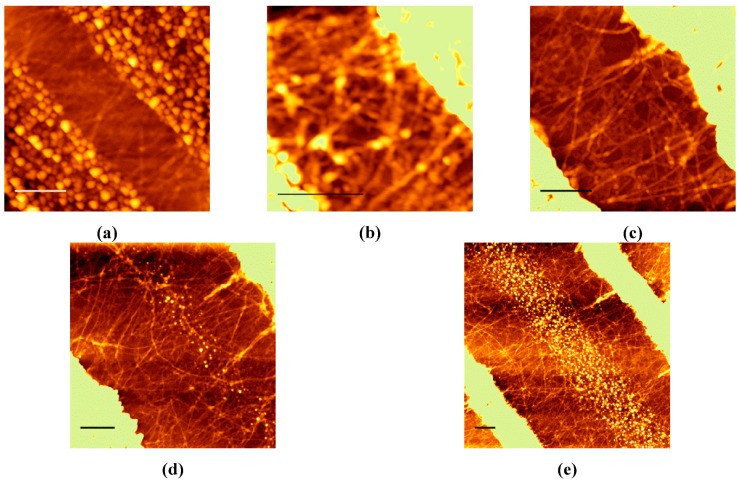
SFM images of nanofibers crystallized in grooves made of CYTOP™ with gap widths of (**a**) 600; (**b**) 900; (**c**) 1500; (**d**) 2400; and (**e**) 3000 nm. The scale bar represents 500 nm.

Nanofibers could be formed in grooves made of CYTOP™ with gap widths of (a) 600; (b) 900; (c) 1500; (d) 2400; and (e) 3000 nm, as shown by the SFM images in Figure 2. In all of the SFM images, the contrast was adjusted to maintain linearity between the tone and height to emphasize the nanofibers, which caused the tone of the tops of the walls on both sides to be overexposed. The surface of the wall was quite rough after RIBE with O_2_ gas to remove the residual layer. In the wider gaps of 2400 and 3000 nm, no obvious uniaxial orientation of the nanofibers was observed**.** On the other hand, the nanofibers formed in narrower gaps of 600, 900, and 1500 nm seemed to be oriented almost perpendicular to the grooves. Nanofibers grown in a narrow groove are generally expected to orient parallel to the groove because nanofibers can grow to more than several micrometers in length in free space, whereas their growth perpendicular to the groove is restricted by the wall. However, the experimental results indicate that the nanofibers did grow perpendicular to the groove, and that their length was limited by the width of the groove.

**Figure 3:**
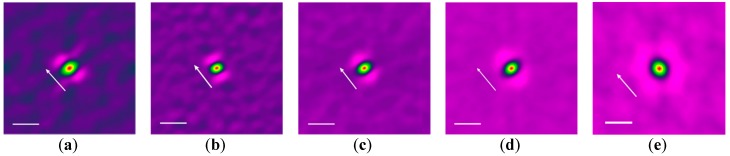
Averaged autocorrelation images of nanofibers crystallized in grooves with gap widths of (**a**) 600; (**b**) 900; (**c**) 1500; (**d**) 2400; and (**e**) 3000 nm. For emphasizing the correlation, the rainbow color was assigned to the correlation function and a strong correlation was assigned to a bright color. The arrow in each image represents the direction of the groove, and the scale bar represents 100 nm.

To precisely evaluate the degree of orientation, averaged autocorrelation images were calculated for various groove widths, as shown in Figure 3. The arrow in each figure represents the direction of the groove in the SFM image. Bright spots symmetrically positioned at the center were clearly observed for gaps with widths of (a) 600; (b) 900; and (c) 1500 nm, which represent the existence of significant orientational correlation; that is, the nanofibers tended to orient uniaxially. The vector between the bright spots and the center corresponds to that perpendicular to the orientation direction; On the other hand, in the averaged autocorrelation images for gap widths of (d) 2400 nm, the bright spot was considerably blurred and broadened, indicating that the uniaxial orientation had become disordered; Furthermore, in the averaged autocorrelation images for a gap width of (e) 3000 nm, there was a fuzzy circle around the center, indicating that the nanofibers were orientationally disordered.

#### 3.1.2. PMMA Grooves

**Figure 4:**
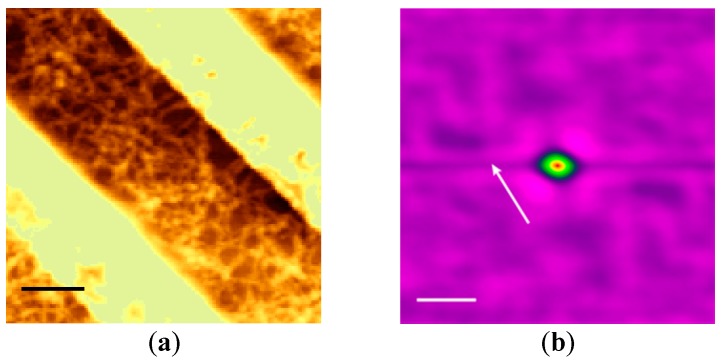
(**a**) SFM image and (**b**) averaged autocorrelation image of nanofibers crystallized in a poly(methyl methacrylate) (PMMA) groove with a gap width of 900 nm. A strong correlation was assigned to a bright color and the arrow represents the direction of the groove in figure (**b**). The scale bars represent (**a**) 500 and (**b**) 100 nm.

To investigate the effect of the groove wall material, we observed the orientation of nanofibers crystallized in grooves made of PMMA with a gap width of 800 nm. Figure 4a shows an SFM image in which the contrast was adjusted to maintain linearity between the tone and height in order to emphasize the nanofibers, which overexposed the top of the groove walls at both sides. The surface of the wall was flatter than those made of CYTOP™. Despite the use of grooves with the same narrow width that led to uniaxially well-oriented nanofibers in CYTOP™ grooves, no uniaxial orientation of nanofibers was observed, and the nanofibers were instead orientationally disordered. In the averaged autocorrelation image shown in Figure 4b, a fuzzy isotropic circle around the center was observed, indicating that the nanofibers were orientationally disordered. According to this result, the PMMA grooves did not cause the nanofibers to independently orient along the width of the groove.

### 3.2. Morphology of Nanofibers on a CYTOP™ Surface

**Figure 5:**
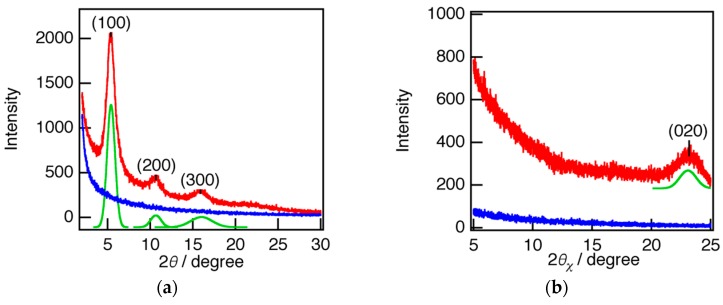
(**a**) Out-of-plane and (**b**) in-plane wide angle X-ray diffraction (WAXD) patterns for nanofibers crystallized on CYTOP™ (red line) and bare CYTOP™ surface without nanofibers (blue line). The diffraction of the nanofibers was fitted by the sum of Lorentzian functions corresponding to each peak component (green line). The baseline used in the fitting function is not shown for simplicity.

To investigate the effect of the CYTOP™ groove wall surface on the nanofiber morphology, P3HT nanofiber crystallized on a CYTOP™ surface without an imprinted groove pattern was studied using WAXD with out-of-plane (θ/2θ) and in-plane (2θ_χ_/φ) displacement. Figure 5a shows the out-of-plane measurements of nanofibers crystallized on CYTOP™ (red line) and bare CYTOP™ surface without nanofibers (blue line). In addition, the diffraction data were fitted by the sum of Lorentzian functions corresponding to each peak component. In the nanofiber diffraction, the larger peak at 2θ ≈ 5.4° indicated a periodic structure with an interlayer distance of the (100) diffraction, *d*_100_ ≈ 16 Å. This is almost the same distance as the sum of twice the molecular length of the tilted side chains in an all-trans conformation and the width of the polythiophene backbone. Furthermore, the higher-order peaks indicated the periodic structure with interlayer distances corresponding to the (200) and (300) diffractions. On the other hand, the broad peak at 2θ ≈ 23° in the in-plane measurements of nanofibers crystallized on CYTOP™ (red line, Figure 5b) indicates (020) diffraction of the polymer chain stacking by π–π interaction between the polymer backbones, *d*_020_ ≈ 3.8 Å. The peak 2θ angles and interlayer distances *d* for the nanofibers crystallized on CYTOP^TM^ and cast on Si after crystallization in solution are summarized in Table 1.

The indices for the nanofibers crystallized on CYTOP^TM^ were almost the same as for those cast on Si after crystallization in solution, which indicates little difference in morphology between the two nanofibers. Furthermore, the observation of indices of (100) and higher order in the out-of-plane measurement and of (020) in the in-plane measurement suggests that the P3HT molecules of the nanofiber have an edge-on arrangement on CYTOP^TM^, which was the same as that for nanofibers cast on Si after crystallization in solution. Therefore, the CYTOP^TM^ surface had no specific effect on the orientation of P3HT nanofibers.

### 3.3. Mechanism of Nanofiber Orientation in the CYTOP^TM^ Groove

Although the CYTOP™ surface itself had no specific effect on the P3HT nanofiber orientation, the nanofibers oriented themselves only in narrow grooves made of CYTOP™. Careful observation of the SFM images shown in Figure 2 indicated that the nanofibers grew from the surface of the CYTOP™ wall. This means that nanofiber nucleation tends to be selectively promoted near the interface of CYTOP™. Furthermore, the growth of the nanofiber perpendicular to the wall is promoted because P3HT molecules are supplied more frequently for nanofiber growth from the direction of the groove center. In wide grooves, nucleation occurred homogeneously within the groove and nanofibers grew in various directions, so that no orientation of the nanofibers was apparent. Similar preferential orientation has been reported for sPS crystallization in AAO cylindrical pores [22]. In this case, nuclei formation at the interface led to preferential orientation with the chain aligned increasingly parallel to the long axis of the pore with decreasing pore diameter, and this effect was superior to the preferential growth direction effect of the confining wall. It is reasonable that the same behavior could occur during the crystallization of P3HT nanofibers. On the other hand, the nucleation of nanofibers occurs everywhere in the groove when using a PMMA wall, which indicates that the PMMA wall has no specific effect on nanofiber nucleation. Nuclei generated around the center of the groove grow isotropically; therefore, preferred orientation was observed when using a PMMA wall.

### 3.4. Conductivity of Nanofibers Oriented in the Groove

**Figure 6:**
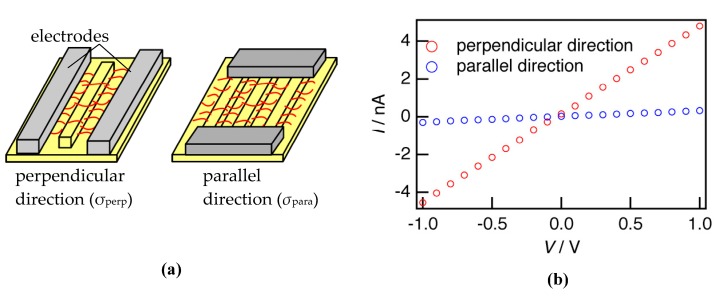
(**a**) Schematic images of conductivity measurements perpendicular (**left**) and parallel (**right**) to the groove and (**b**) *I*–*V* characteristics perpendicular and parallel to the groove.

The uniaxial orientation achieved in a narrow groove can lead to anisotropic electric conduction, which is the purpose of this study. The anisotropic conductivity of nanofibers oriented in the groove was measured by depositing electrodes parallel (to measure the conductivity perpendicular to the groove) and perpendicular (to measure the conductivity parallel to the groove) to the groove. Schematic images of the electrode placement are shown in Figure 6a, and the obtained *I*–*V* characteristics are shown in Figure 6b. The conductivities perpendicular (σ_perp_) and parallel (σ_para_) to the groove were estimated to be 1.61 × 10^−4^ and 8.45 × 10^−6^ S·cm^−1^, respectively, where the width and length of the conductor region contributing to the conductivity corresponded approximately to the width and gap of the electrodes, and the thickness was estimated as an average from SFM images of nanofibers. σ_perp_, directed along the oriented nanofiber, was more than an order of magnitude larger than σ_para_, perpendicular to the oriented nanofiber. Although there are groove walls, which obviously hinder conduction perpendicular to the groove, σ_perp_ was much larger than σ_para_, which indicates that anisotropic conduction was achieved by the controlled orientation of the nanofibers.

## 4. Conclusions

The crystallization of whisker-type P3HT nanofibers in a narrow groove was shown to be an effective method of aligning the nanofibers. This was suggested to result from the selective promotion of nucleation near the interface of CYTOP™ with fluoro-groups. This method of orienting nanofibers was successfully used to achieve anisotropic conduction with a greater than ten-fold difference in conductivity between the directions parallel and perpendicular to the aligned fibers.

## Figures and Tables

**Table 1:** Peak angle 2θ and interlayer distance *d* of nanofibers crystallized on CYTOP™ and cast on Si after nanofiber formation in solution.

Sample	2θ_100_/deg.	*d*_100_/Å	2*θ*_020_/deg.	*d*_020_/Å
Nanofiber on CYTOP™	5.41	16.3	23.09	3.85
Casted Nanofiber	5.23	16.9	23.47	3.79
